# Destabilization of Indomethacin-Paracetamol Co-Amorphous Systems by Mechanical Stress

**DOI:** 10.3390/pharmaceutics16010067

**Published:** 2023-12-31

**Authors:** Rong Di, Thomas Rades, Holger Grohganz

**Affiliations:** Department of Pharmacy, University of Copenhagen, 2200 Copenhagen, Denmark; rong.di@sund.ku.dk (R.D.); holger.grohganz@sund.ku.dk (H.G.)

**Keywords:** grinding, co-amorphous system, quench-cooling, thermal analysis, eutectic behavior, optimal mixing ratio

## Abstract

Using co-amorphous systems (CAMS) has shown promise in addressing the challenges associated with poorly water-soluble drugs. Quench-cooling is a commonly used CAMS preparation method, often followed by grinding or milling to achieve a fine powder that is suitable for subsequent characterization or further down-stream manufacturing. However, the impact of mechanical stress applied to CAMS has received little attention. In this study, the influence of mechanical stress on indomethacin—paracetamol CAMS was investigated. The investigation involved thermal analysis and solid-state characterization across various CAMS mixing ratios and levels of mechanical stress. The study revealed a negative effect of mechanical stress on stability, particularly on the excess components in CAMS. Higher levels of mechanical stress were observed to induce phase separation or recrystallization. Notably, samples at the optimal mixing ratio demonstrated greater resistance to the destabilization caused by mechanical stress. These results showed the significance of careful consideration of processing methods during formulation and the significance of optimizing mixing ratios in CAMS.

## 1. Introduction

Improving the solubility of poorly water-soluble drugs has been one of the most critical problems to solve in the pharmaceutical development of low-molecular-weight drugs for many decades. Multiple methods have been developed to address this issue [1,2]. Co-amorphous systems (CAMS) have gradually developed to be a promising and feasible solution to this problem [3]. CAMS contains a low-molecular-weight carrier (termed the co-former) to stabilize and deliver the drug in its amorphous form, which is a high-energy solid state (and thus thermodynamically unstable) but with a higher (apparent) water solubility compared to the respective crystalline forms [4]. The small-molecule co-former can be an amino acid [5], a drug [6,7], or an organic acid [8].

Choosing a suitable mixing ratio between the drug and the co-former plays an important role in the formation of successful CAMS [9]. Earlier research demonstrated that employing an optimal mixing ratio is beneficial for the physical stability and dissolution performance of CAMS [10,11]. However, across all studies on CAMS up to 2021, only 5.8% of them included molar ratio optimization [3]. The importance of mixing ratio optimization has been largely overlooked. The eutectic point of binary systems was found to be highly related to the optimal mixing ratios of the respective CAMS [12]. It has been reported that two components that can form an eutectic mixture at this mixing ratio may also form the strongest molecular interactions as the respective CAMS compared to other mixing ratios [13]. Stronger molecular interactions within the CAMS in turn are highly related to higher physical stability [10,11]. Therefore, detection of the eutectic point can be used as a tool to find the optimal mixing ratio of a CAMS. Yet, even with a suitable (“optimal”) mixing ratio found, there are still additional aspects to consider.

The preparation method is one such aspect to consider when preparing CAMS. The preparation of CAMS can be carried out in two fundamentally different ways: the kinetic pathway (e.g., mechanical activation [10]) and the thermodynamic pathway (e.g., solvent-evaporation [11] and quench-cooling [14]). Quench-cooling is a commonly used technique to prepare CAMS, with approximately 48.8% of the studies following the thermodynamic pathway having used this method [3]. Using quench-cooling or hot-melt extrusion as preparation methods, a subsequent grinding or milling process is commonly performed to obtain finely powdered CAMS for later characterization or downstream manufacturing [15,16]. Despite the use of grinding in the preparation of CAMS, a dedicated investigation into the consequences of grinding for co-amorphous samples is missing. The aim of this study is to investigate the influence of mechanical stress, caused by grinding, on CAMS upon mixing ratio optimization.

It is established that milling can gradually introduce defects in crystals and potentially lead to amorphization [17,18]. Several studies have investigated the effect of mechanical stress on amorphous materials [19]. Bhugra et al. [20] discovered that even minor mechanical stress applied to amorphous indomethacin (IND) significantly changed its thermal behavior. Additionally, a study conducted by Yang et al. [21] revealed that milling of hot melt extruded solid dispersions enhanced molecular mobility, accelerated phase separation, and induced recrystallization upon additional heating. Furthermore, the authors found that amorphous solid dispersions, with drug loadings within the drug’s polymer solubility limit, experience less influence from mechanical stress, whereas supersaturated amorphous dispersions exhibit a greater influence of mechanical stress on phase separation behavior. Few studies [22] have investigated the impact of mechanical stress, resulting from compaction, on CAMS during downstreaming. Surprisingly, to the knowledge of the authors, there has been no investigation into the influence of mechanical stress resulting from grinding on CAMS.

In this study, the influence of mechanical stress on the stability of CAMS was investigated. IND and paracetamol (PAR) were chosen as model drugs to form the drug-drug CAMS. Firstly, the eutectic behavior of the IND–PAR mixtures was studied for mixing ratio optimization. Subsequently, CAMS of different mixing ratios were subjected to mechanical stress through grinding and later investigated by differential scanning calorimetry (DSC) and variable-temperature X-ray powder diffraction (VT-XRPD). The results showed that mechanical stress has a negative effect on the stability of CAMS, as it accelerates phase separation and triggers recrystallization. Optimizing the mixing ratio in CAMS not only enhances stability during storage but also leads to higher resistance against instability caused by mechanical stress. This research emphasized the significance of careful consideration of the processing method during formulation and the importance of mixing ratio optimization in CAMS.

## 2. Materials and Methods

### 2.1. Materials

Paracetamol (PAR) (molecular weight: 151.163 g/mol, CSD: HXACAN01) was purchased from Fargon Service B.V. (Uitgeest, The Netherlands). Indomethacin (IND) (molecular weight: 357.787 g/mol, CSD: INDMET03) was purchased from Hovione Farmaciência S.A. (Loures, Portugal). Both drugs were used as received.

### 2.2. Methods

#### 2.2.1. Sample Preparation

Crystalline physical mixtures (PM) of IND–PAR at different mixing ratios (from 90% IND–10% PAR to 10% IND–90% PAR (mol/mol), in steps of 10%) were prepared by gently mixing the crystalline forms of the two drugs (1 g in total) using a mortar and pestle.

CAMS were prepared by quench-cooling. The PM were placed on aluminum foil over a preheated hot plate (180 °C) until the PM fully melted (visual confirmation). The molten samples were cooled by placing the foil (with molten samples on top) on a stainless steel block (stored at around 5 °C). Pure amorphous IND and amorphous PAR were prepared by the same quench-cooling method with the same heating and cooling temperatures.

Mechanical stress was applied to the samples by grinding the CAMS with a mortar and pestle. To study the influence of different degrees of mechanical stress on the CAMS, different grinding durations were applied (0.5 min, 1 min, 5 min, 10 min, 20 min, and 30 min) to obtain different ground CAMS (G-CAMS).

#### 2.2.2. Thermal Analysis

The thermal behavior of the samples was analyzed using a Discovery DSC (TA Instruments, New Castle, DE, USA). All measurements were conducted under a constant 50 mL/min nitrogen gas flow. An amount of 2–5 mg of sample were weighed into aluminum pans and sealed with pierced lids. To measure the eutectic behavior of the samples, crystalline IND, crystalline PAR, and the PM of IND–PAR at different mixing ratios were heated up from 25 °C to 180 °C (at a rate of 10 °C/min to achieve better resolution). All measurements for the eutectic behavior investigation were conducted in triplicates. To investigate the thermal behavior of the CAMS or G-CAMS, samples were equilibrated at −20 °C for 1 min, then heated up to 180 °C (at a rate of 20 °C/min for better sensitivity). The DSC data was collected and analyzed using Trios software (version 5.1.1, TA Instruments, New Castle, DE, USA).

The water content of the samples was assessed by using thermogravimetric analysis (TGA) (Discovery TGA, TA Instruments, New Castle, DE, USA). Samples (around 10 mg) were heated from 25 °C to 300 °C at a rate of 20 °C/min. All measurements were conducted under a constant 50 mL/min nitrogen gas flow. Data was collected and analyzed using Trios software (version 5.1.1, TA Instruments, New Castle, DE, USA).

#### 2.2.3. XRPD and VT-XRPD

The solid state of the samples was investigated with an X’Pert PRO diffractometer (PANalytical, Almelo, The Netherlands), equipped with a copper anode (Cu Kα radiation, λ = 1.54187 Å). The generator voltage was 45 kV, and the tube current was 40 mA. Samples were scanned from 5° to 35° 2θ in reflection mode, with a scan rate of 0.067° 2θ/s and a step size of 0.026° 2θ. A Bragg–Brentano parafocusing geometry was used.

For VT-XRPD measurements, an Anton Paar CHC sample stage (Anton Paar GmbH, Graz, Austria) was mounted to the diffractometer. To investigate the eutectic behavior, crystalline IND, crystalline PAR, and PM with various mixing ratios (from 10% IND–90% PAR to 90% IND–10% PAR) were scanned at 25 °C and from 120 to 170 °C at 5 °C intervals. To investigate the thermal behavior of the CAMS and G-CAMS, samples were scanned from 30 to 170 °C at 10 °C intervals. Before each scan, a temperature equilibrium step was carried out for 2 min. XRPD data was collected and analyzed using X’Pert Data Collector and X’Pert Highscore Plus software (version 2.2.4, PANalytical, Almelo, The Netherlands), respectively.

#### 2.2.4. Physical Stability Test

Physical stability tests were performed on amorphous IND, amorphous PAR, IND–PAR CAMS, and G-CAMS. The samples were stored under dry conditions (0% relative humidity using phosphorus pentoxide) at room temperature. Samples were measured by XRPD weekly until recrystallization was detected.

#### 2.2.5. Construction of Phase Diagram and Tammann Diagram for Eutectic Mixtures

For the phase diagram, the experimental melting temperatures (T_m_) of IND–PAR PM were obtained from the DSC experiments. From the DSC graphs of IND–PAR PM at different mixing ratios, the onset temperature of the first melting behavior (corresponding to the eutectic temperature (T_e_)) and the peak temperature of the second melting behavior (corresponding to the liquidus temperature (T_l_)) were obtained to construct the phase diagram. The theoretical T_m_ of IND–PAR PM with different mixing ratios was calculated from the simplified Schröder–Van Laar Equation [12,23].

$$In\left(X\right)=\frac{∆H_{0}}{R}\left(\frac{1}{T_{0}}-\frac{1}{T}\right)$$

where ∆H_0_ represents the heat of fusion (J·mol^−1^) and T_0_ represents the T_m_ (in Kelvin) of one of the pure drugs in the mixture. T is the T_m_ of the binary mixture at a specific molar ratio, X is the molar ratio, and R is the gas constant (8.314 J·K^−1^·mol^−1^).

For the Tammann diagram, the enthalpy changes were obtained from DSC experiments.

## 3. Results and Discussion

### 3.1. Eutectic Behavior Investigation on IND–PAR Systems

#### 3.1.1. Eutectic Behavior of IND–PAR Systems Investigated by DSC

**Figure 1:**
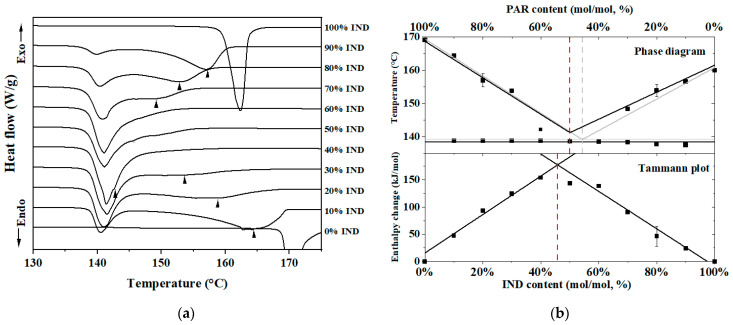
(**a**) DSC thermograms of crystalline IND, crystalline PAR and crystalline IND–PAR PM at different mixing ratios (from 90% IND–10% PAR to 10% IND–90% PAR (mol/mol)). Black arrows indicate T_l_; (**b**) phase diagram and Tammann plot of IND–PAR binary system. Grey solid lines represent the theoretical data, dashed lines indicate the T_e_ from theoretical data (grey) and experimental data (red), and the black squares represent experimental data.

DSC was used to investigate the eutectic behavior of the IND–PAR systems for mixing ratio optimization. Figure 1a shows the thermograms of crystalline IND, crystalline PAR, and their PM at different mixing ratios. The T_m_ of IND and PAR were determined to be 160 °C and 169 °C, respectively. Melting endotherms at around 138 °C were observed in all PM, and this temperature remained consistent regardless of the IND–PAR PM molar ratios. This observed T_m_ can be attributed to T_e_ [24]. With the exception of samples containing 60% IND–40% PAR and 50% IND–50% PAR, most of the PM exhibited a second melting endotherm ranging from 169 °C to 138 °C. This T_m_ can be attributed to T_l_, which represents the melting of the excess components, and it varies depending on the mixing ratios [24].

A phase diagram and Tammann plot (Figure 1b) were constructed to obtain the eutectic point. From the phase diagram, the eutectic point was determined at 54% IND–46% PAR and 50% IND–50% PAR from the theoretical data and experimental data, respectively. From the Tammann plot, the eutectic point was determined at 46% IND–54% PAR.

#### 3.1.2. Eutectic Behavior of IND–PAR Systems Investigated by VT-XRPD

**Figure 2:**
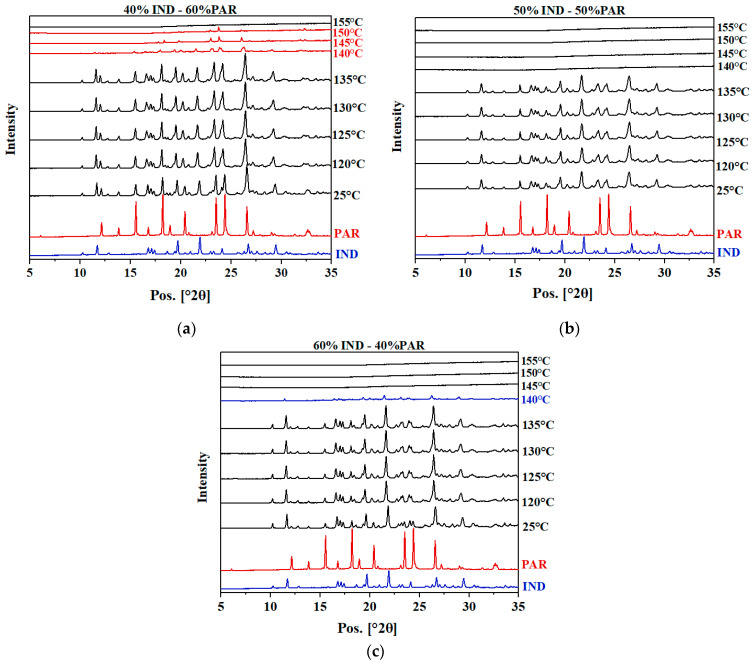
XRPD diffractograms of crystalline PM with (**a**) 40% IND–60% PAR, (**b**) 50% IND–50% PAR, and (**c**) 60% IND–40% PAR (mol/mol) at 25 °C and 120 to 170 °C (with 5 °C intervals). The XRPD diffractograms of crystalline IND and crystalline PAR are also shown in each figure. Diffractograms above 155 °C showed identical patterns and were excluded from the graph.

To validate the eutectic point, the solid state of the IND–PAR systems at varying temperatures was investigated by VT-XRPD. Results for samples with 40% IND–60% PAR, 50% IND–50% PAR, and 60% IND–40% PAR are shown in Figure 2. These mixtures were chosen due to the absence of clear T_l_ in the DSC results. Results for other mixing ratios can be found in Supplementary Figure S1.

The diffractograms of crystalline PM with various mixing ratios showed similarities at temperatures below 140 °C. From 25 to 135 °C, reflections attributed to both IND and PAR were observed. Between 135 and 140 °C, there was a substantial decrease in the intensities of the reflections, indicating melting. Therefore, the T_e_ was identified in the range of 135 to 140 °C, which aligns well with the T_e_ value obtained from DSC results (138 °C). Above the T_e_, different thermal behaviors were observed for the different samples. For the 40% IND–60% PAR sample, reflections belonging to PAR can still be observed, which means the sample contained excess PAR. For the sample with 50% IND–50% PAR, all reflections vanished simultaneously at T_e_, confirming that the eutectic point is at 50% IND–50% PAR. For the sample with 60% IND–40% PAR, reflections attributed to IND were still noticeable at 140 °C, indicating the presence of excess IND.

Overall, the VT-XRPD measurements confirmed the position of the eutectic point at 50% IND–50% PAR.

### 3.2. Influence of Mixing Ratios on CAMS Stability

Based on the eutectic behavior investigation of IND–PAR systems, the composition of 50% IND–50% PAR is considered the eutectic mixture for the IND–PAR binary system. To test the alignment between the eutectic mixture of the PM and the optimal mixing ratio of the corresponding CAMS, a stability test was carried out on both neat amorphous drugs and the CAMS with various mixing ratios (ranging from 10% IND–90% PAR to 90% IND–10% PAR).

Table 1 shows the stability results of all CAMS and neat amorphous drugs. Upon preparation, all samples aside from PAR were amorphous, and the T_g_ of the corresponding mixtures followed the theoretical expectations based on the Gordon-Taylor equation, indicating a homogenous system without notable interactions (see Supplementary Figure S2). All samples were recrystallized during storage. For the 40% IND–60% PAR system, crystalline reflections attributed to both IND and PAR were observed, while in all other cases, the reflections could be assigned to the more prevalent component (“excess component”). Notably, the 50% IND–50% PAR sample exhibited the highest stability and remained amorphous for 9 weeks. Hence, the stability study confirmed that the eutectic mixture of 50% IND–50% PAR is the optimal mixing ratio for CAMS.

### 3.3. Influence of Grinding on CAMS Stability—Investigated by Long-Term Stability Test and TGA

**Figure 3:**
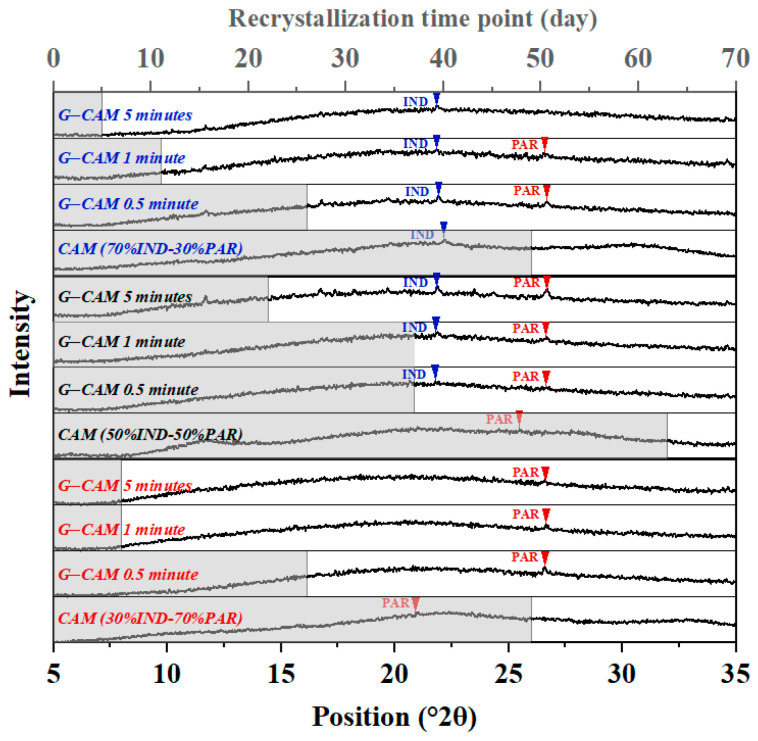
Stability test results of CAMS and G-CAMS after various grinding times (0.5, 1, and 5 min). XRPD patterns of the samples after recrystallization are also shown in the figure. The gray bars indicate the time period during which the sample remained in its amorphous form.

The most stable mixing ratio for the IND–PAR CAMS was found to be at 50% IND–50% PAR (within the precision of the mixing steps chosen). However, it remains unclear how mechanical stress might affect the stability of CAMS with varying mixing ratios. To address this, a physical stability test was carried out on both CAMS and G-CAMS with three different mixing ratios: samples with excess IND (70% IND–30% PAR), samples at the optimal mixing ratio (50% IND–50% PAR), and samples with excess PAR (30% IND–70% PAR). These G-CAMS were subjected to different durations of grinding (0.5 min, 1 min, 5 min) to apply different degrees of mechanical stress. The results are shown in Figure 3.

All samples showed signs of recrystallization during the stability test. G-CAMS exhibited significantly lower physical stability in comparison to their respective CAMS, even with minimal grinding time. Furthermore, G-CAMS, subjected to longer grinding times, experienced earlier recrystallization. Among these, at the same grinding times, G-CAMS at the optimal mixing ratio (50% IND–50% PAR) proved to be the most stable, compared to G-CAMS with excess components. During the stability test, both IND and PAR recrystallized in G-CAMS with 50% IND–50% PAR and with 70% IND–30% PAR. In G-CAMS with 30% IND–70% PAR, only PAR is recrystallized.

Previous studies have indicated that grinding may enhance the water absorption of a material [25]. Water often acts as a plasticizer in amorphous formulations, and its plasticizing effect can potentially destabilize the CAMS [26,27]. TGA tests were thus conducted to quantify the water content in both CAMS and G-CAMS. The results are detailed in Table 2. The TGA graphs can be found in Supplementary Figure S4.

Based on the TGA results, G-CAMS showed elevated water content compared to CAMS, indicating that grinding introduced moisture into the samples. This elevated water content could be expected to partially contribute to the destabilization of G-CAMS. However, G-CAMS at different grinding times showed different stability according to the previous long-term stability test results, despite a similar water content. Therefore, it can be concluded that water is not a major factor in the destabilization of the G-CAMS.

### 3.4. Influence of Grinding on CAMS with Different Mixing Ratios—Investigated by DSC and VT-XRPD

DSC and VT-XRPD measurements were conducted on both CAMS and G-CAMS (ground for 0.5 min) to further investigate how grinding affects the CAM samples. The DSC and XRPD results for ground-amorphous IND and ground-amorphous PAR can be found in Supplementary Figure S5.

#### 3.4.1. Influence of Grinding on Samples Containing Excess Components

**Figure 4:**
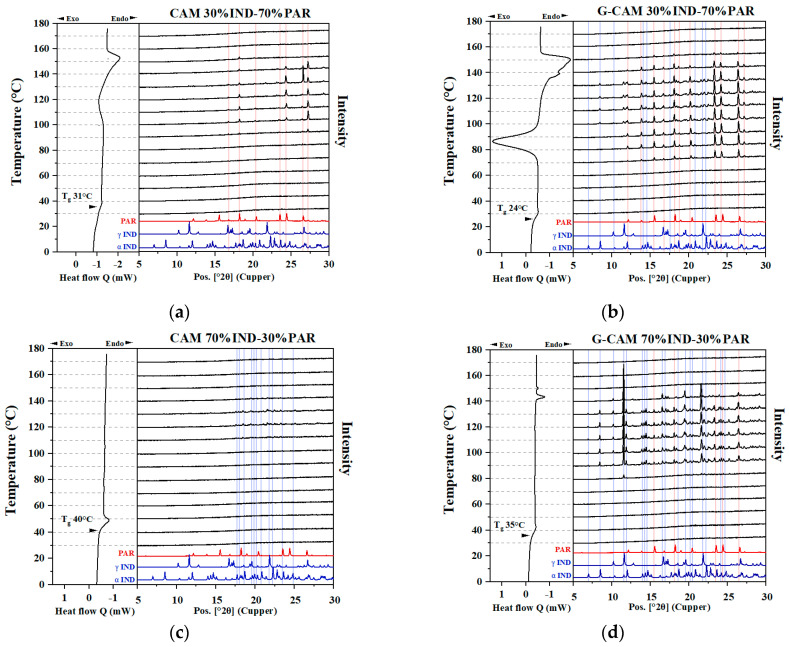
DSC and VT-XRPD results on (**a**) 30% IND–70% PAR CAMS, (**b**) 30% IND–70% PAR G-CAMS, (**c**) 70% IND–30% PAR CAMS, and (**d**) 70% IND–30% PAR G-CAMS. Red and blue solid lines represent the positions of the reflections corresponding to crystalline PAR and crystalline IND, respectively.

For CAMS containing excess PAR (30% IND–70% PAR) (Figure 4a), a recrystallization peak was observed in the DSC (around 100 °C) and identified to be PAR based on the presence of characteristic crystalline reflections in the VT-XRPD measurements. This is due to the existence of excess PAR in the sample, in line with previous studies that found that the excess component has a higher tendency to recrystallize [10]. The recrystallized excess PAR melted at approximately 152 °C, aligning with the T_l_ for the 30% IND–70% PAR PM. Similarly, in CAMS containing excess IND (70% IND–30% PAR) (Figure 4c), the recrystallization and melting of the excess IND were observed in the VT-XRPD. However, this phenomenon was not observed by the DSC, possibly due to the limited sensitivity of the DSC or the ambient conditions during the VT-XRPD experiment.

A rather different picture was obtained for the G-CAMS. For 30% IND–70% PAR G-CAMS (Figure 4b), the T_g_ decreased from 31 °C to 24 °C compared to the respective CAMS, which can be explained by the plasticizing effect of water introduced during grinding. A new melting endotherm was found at around 138 °C, corresponding to the T_e_ of the IND–PAR system. This finding indicates the recrystallization of both IND and PAR, resulting in the formation of the eutectic mixture that melted at T_e_. The VT-XRPD results revealed the same conclusion, as crystalline reflections belonging to IND and PAR were observed within the temperature range of 80 to 130 °C. Similarly, for 70% IND–30% PAR G-CAMS (Figure 4d), the T_g_ shifted from 40 °C to 35 °C due to increased water content. Two melting peaks were detected at around 138 °C and 150 °C aligned with the T_e_ and the T_l_ of the 70% IND–30% PAR PM, respectively, indicating the recrystallization of both excess IND and PAR supported by VT-XRPD results.

Interestingly, amorphous IND recrystallized to the α form in CAMS, while upon grinding, it recrystallized to both γ and α forms during the DSC runs. This finding aligns with prior research, which discussed that a rapid quench cooling rate can result in higher local disorder in the final amorphous IND, potentially leading to recrystallization towards the α form [28]. In contrast, amorphous IND prepared with a slow quench cooling rate may favor recrystallizing to the γ form [28]. Additionally, the finding suggests that under low milling intensity, amorphous IND tended to recrystallize to the γ form [28].

#### 3.4.2. Influence of Grinding on Samples with Optimal Mixing Ratio

**Figure 5:**
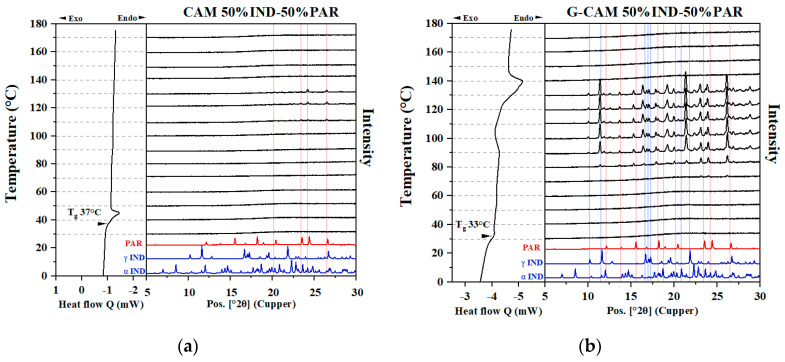
DSC and VT-XRPD results for (**a**) CAMS and (**b**) G-CAMS at the mixing ratio 50% IND–50% PAR. Red and blue solid lines represent the positions of the reflections corresponding to crystalline PAR and crystalline IND, respectively.

No recrystallization or melting events were observed for the 50% IND–50% PAR CAMS (Figure 5a) in the DSC thermograms. However, small crystalline reflections attributed to PAR were observed in the VT-XRPD diffractograms at 120 and 130 °C. This might indicate the possible existence of a small amount of excess PAR in the 50% IND–50% PAR CAMS.

As indicated in Figure 5b, a decrease in T_g_ (from 37 to 33 °C) was observed due to the increased water content after grinding. Two recrystallization peaks appeared at 90 °C and 120 °C, corresponding to PAR and IND, respectively, according to VT-XRPD. A single melting peak was observed at around T_e_ (138 °C), indicating that recrystallization of the eutectic mixture consisted of IND and PAR.

Overall, in CAMS with excess components, the excess component demonstrated a higher tendency to recrystallize compared with the other component in the CAMS. The application of even minor mechanical stress could destabilize CAMS, leading not only to the recrystallization of excess components but also to the recrystallization of initially stable components upon heating.

### 3.5. Influence of Different Grinding Times on CAMS with Different Ratios—Investigated by DSC and XRPD

The influence of different grinding times on CAMS was investigated by DSC and XRPD measurements of G-CAMS (ground for 0.5, 1, 5, 10, 20, and 30 min).

#### 3.5.1. Samples Containing Excess PAR (30% IND–70% PAR)

**Figure 6:**
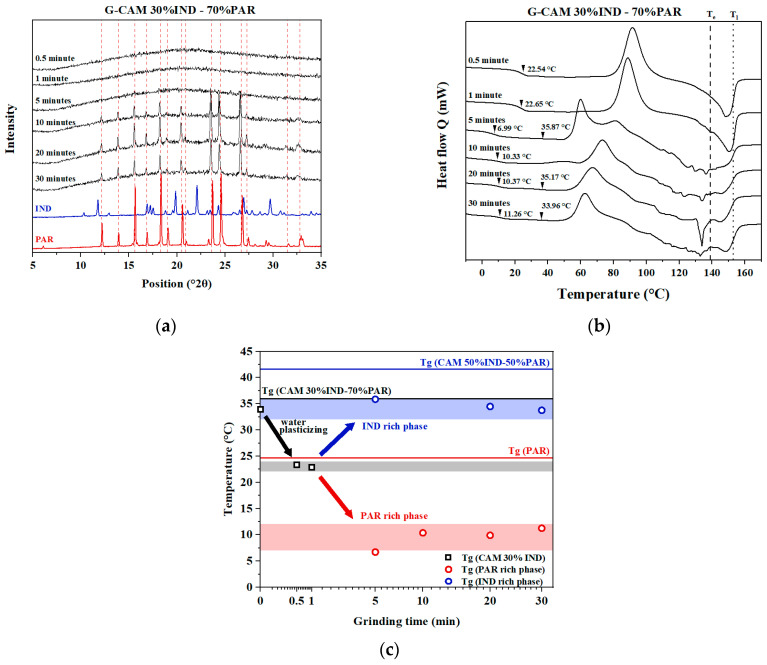
(**a**) XRPD diffractograms of crystalline IND, crystalline PAR, and the G-CAMS containing 30% IND–70% PAR. The red dashed lines represent the position of crystalline peaks corresponding to PAR; (**b**) DSC thermograms of the G-CAMS containing 30% IND–70% PAR. The black dashed and dotted lines show the T_e_ and the T_l_, respectively, of the sample with 30% IND–70% PAR; (**c**) T_g_ distribution graph for 30% IND–70% PAR CAM and the respective G-CAMS.

According to the results presented in Figure 6a, the XRPD diffractograms of G-CAMS ground for 0.5 and 1 min were both amorphous. However, the corresponding DSC results (Figure 6b) showed that longer grinding times led to earlier recrystallization events. Moreover, for G-CAMS ground for 5 min or more, crystalline reflections attributed to PAR were observed in the XRPD, which indicates recrystallization of excess PAR during the grinding. The DSC results further revealed degradation during the runs, manifested as fluctuations in the DSC curves when temperatures exceeded 100 °C. Notably, in some G-CAMS, the presence of two distinct T_g_ was observed, which indicated a potential phase separation.

To better observe the potential phase separation, a graph illustrating grinding time against temperature was constructed (Figure 6c). In this figure, the T_g_ values of G-CAMS with varying grinding times are presented as data points. With short grinding durations (0.5 or 1 min), only one T_g_ was observed at around 24 °C, matching the T_g_ of 30% IND-70% PAR CAMS with increased water content from grinding. With extended grinding, two distinct T_g_ (10 and 33 °C) were observed, which indicated two phases in the systems. The two phases can be distinguished as the IND-rich phase (T_g_ 33 °C) and the PAR-rich phase (T_g_ 10 °C). The T_g_ value (33 °C) of the IND-rich phase aligns with the T_g_ of the 50% IND-50% PAR CAMS, which is the optimal mixing ratio with water. The T_g_ value (10 °C) of the PAR-rich phase corresponds to the T_g_ of pure amorphous PAR with water. To validate the phase separation behavior, the experiments were replicated, and the results are presented in Supplementary Figure S6. Previous studies have also reported phase separation in IND–PAR CAMS with aging [29,30].

#### 3.5.2. Samples with Optimal Mixing Ratio (50% IND–50% PAR)

**Figure 7:**
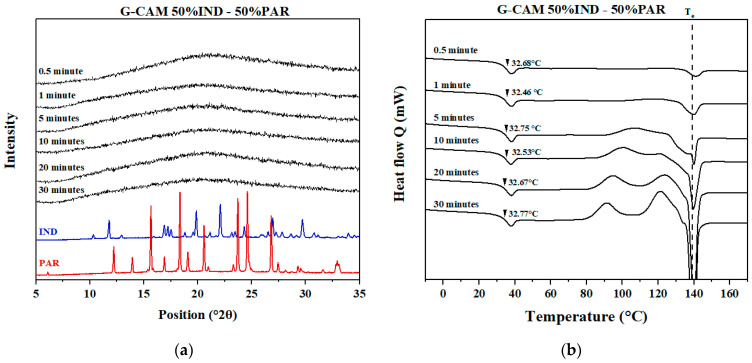
(**a**) XRPD diffractograms of crystalline IND, crystalline PAR, and the G-CAMS containing 50% IND–50% PAR; (**b**) DSC thermograms of the G-CAMS containing 50% IND–50% PAR. The black dashed line shows the T_e_ of the sample with 50% IND–50% PAR.

The XRPD results (Figure 7a) for G-CAMS, regardless of grinding duration, showed an amorphous form, indicating no recrystallization even after 30 min of grinding. Conversely, the DSC results (Figure 7b) highlighted the influence of grinding duration on the thermal behavior of the samples. Longer grinding times led to recrystallization occurring at lower temperatures and in larger quantities, as evident from the enthalpy of the recrystallization and melting peaks. However, regardless of grinding time, only one single melting peak at T_e_ (138 °C) was observed, indicating the recrystallization of the eutectic mixture (50% IND–50% PAR). The T_g_ remained stable at around 33 °C across all samples at different times.

#### 3.5.3. Samples Containing Excess IND (70% IND–30% PAR)

**Figure 8:**
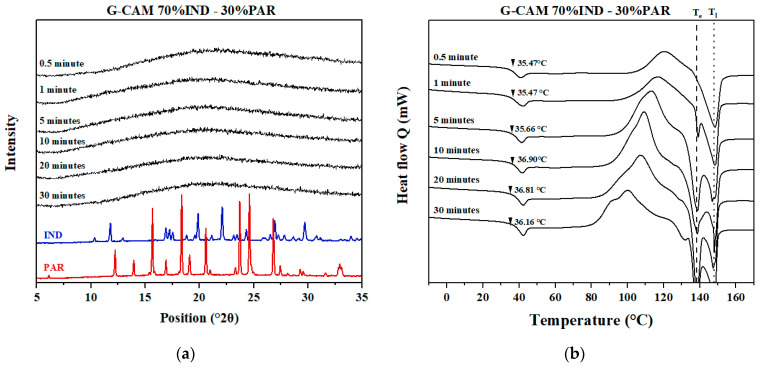
(**a**) XRPD diffractograms of crystalline IND, crystalline PAR, and the G-CAMS containing 70% IND–30% PAR; (**b**) DSC thermograms of the G-CAMS containing 70% IND–30% PAR. The black dashed and dotted lines show the T_e_ and the T_l_, respectively, of the sample with 70% IND–30% PAR.

Similar to the XRPD results of 50% IND–50% PAR G-CAMS, the influence of grinding time on CAMS cannot be observed with XRPD (Figure 8a). However, differences can be observed by DSC (Figure 8b). Recrystallization occurred at a lower temperature with longer grinding times, indicating a decrease in stability. Interestingly, with longer grinding times, the enthalpy of the melting peak at T_e_ (138 °C) kept increasing, while the enthalpy of the melting peak at T_l_ (148 °C) stayed relatively unchanged. This suggests that with longer grinding times, a greater amount of the IND–PAR eutectic mixture recrystallized. In contrast, longer grinding times had no significant influence on the amount of recrystallized excess IND. The T_g_ did not show significant differences among the different G-CAMS.

Overall, an increased level of mechanical stress resulted in a greater degree of instability in CAMS. However, with the same level of mechanical stress, the sample with the optimal mixing ratio consistently showed a lower recrystallization tendency compared to samples with excess components.

## 4. Conclusions

IND–PAR systems exhibited eutectic behavior, with the eutectic mixture identified at a 50% IND–50% PAR (mol/mol) ratio. The eutectic point was further confirmed as the optimal mixing ratio to prepare the most stable CAMS. Mechanical stress was found to have a negative effect on the stability of CAMS, as it accelerated phase separation and triggered recrystallization. The degree of destabilization increased with increasing levels of mechanical stress. For CAMS with excess components, mechanical stress had a greater impact on the excess component, causing it to recrystallize before the other component. CAMS at an optimal mixing ratio showed higher stability under mechanical stress. This suggests that optimizing the mixing ratio in CAMS not only enhances stability during storage but also leads to higher resistance against instability caused by mechanical stress.

## Figures and Tables

**Table 1:** Stability results for amorphous IND, amorphous PAR, and IND–PAR CAMS with different mixing ratios (from 90% IND–10% PAR to 10% IND–90% PAR (mol/mol)). The XRPD diffractograms of the samples at the time point of first observation of recrystallization can be found in Supplementary Figure S3.

Sample Composition	Recrystallization Time	Recrystallized Component
Amorphous IND	4 weeks	IND
90% IND–10% PAR	4 weeks	IND
80% IND–20% PAR	7 weeks	IND
70% IND–30% PAR	7 weeks	IND
60% IND–40% PAR	6 weeks	IND
50% IND–50% PAR	9 weeks	IND
40% IND–60% PAR	7 weeks	IND and PAR
30% IND–70% PAR	7 weeks	PAR
20% IND–80% PAR	2 weeks	PAR
10% IND–90% PAR	1 week	PAR
Amorphous PAR	0 week	PAR

**Table 2:** Water contents of CAMS and G-CAMS after various grinding times (0.5, 1, and 5 min). The water content is presented as the weight ratio of the samples. The water content of 50% IND–50% PAR was not measured. However, being prepared via quench-cooling implies that it is likely to also have a low water content, similar to the other mixing ratios.

Sample Compositions	CAMS (0 min)	G-CAMS (0.5 min)	G-CAMS (1 min)	G-CAMS (5 min)
30% IND–70% PAR	0.02%	0.25%	0.33%	0.34%
50% IND–50% PAR	-	0.35%	0.28%	0.34%
70% IND–30% PAR	0.00%	0.22%	0.30%	0.28%

## Data Availability

The data presented in this study are available on request from the corresponding author.

## Supplementary Materials

The following supporting information can be downloaded at: https://www.mdpi.com/article/10.3390/pharmaceutics16010067/s1, Figure S1: The VT-XRPD diffractograms of IND–PAR PM with different ratios; Figure S2: Comparison of the theoretical T_g_ (calculated with the Gordon–Taylor equation) and the experimental T_g_ (obtained from DSC results) of IND–PAR co-amorphous systems at different mixing ratios (from 90% IND–10% PAR to 10% IND–90% PAR (mol/mol)). The T_g_ of amorphous IND and amorphous PAR were obtained from DSC measurements; Figure S3: Stability test results of IND–PAR CAMS with various mixing ratios; Figure S4: TGA results of CAMS and G-CAMS with different mixing ratios; Figure S5: DSC and XRPD results of ground amorphous IND and ground amorphous PAR; Figure S6: DSC graphs of 30% IND–70% PAR G-CAMS.
