# Supplementary material for: Destabilization of Indomethacin-Paracetamol Co-Amorphous Systems by Mechanical Stress
Source: Pharmaceutics. 2023 Dec 31;16(1):67. doi: 10.3390/pharmaceutics16010067 (PMC10818836; doi:10.3390/pharmaceutics16010067)
Supplement: Supplementary file 1 [file pharmaceutics-16-00067-s001.zip › pharmaceutics-2752081-supplementary.pdf]

Supplementary Materials: Destabilization of Indomethacin-Paracetamol Co-Amorphous Systems by Mechanical Stress

Rong Di, Thomas Rades * and Holger Grohganz

Figure S1. VT-XRPD diffractograms of IND – PAR PM with different drug ratios.

(10% IND – 90% PAR).

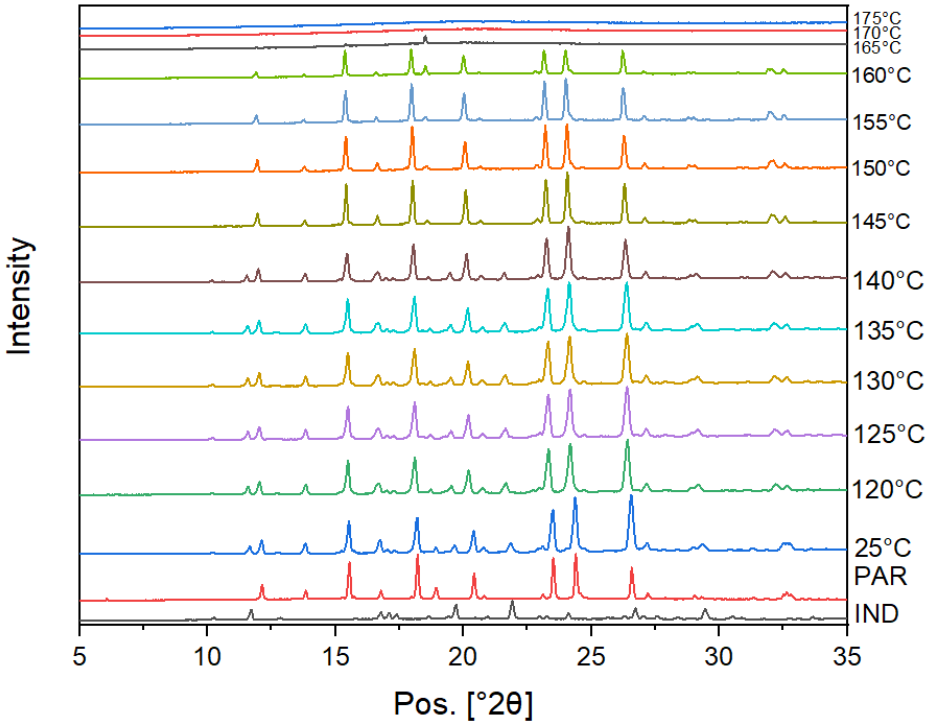

20% IND – 80% PAR

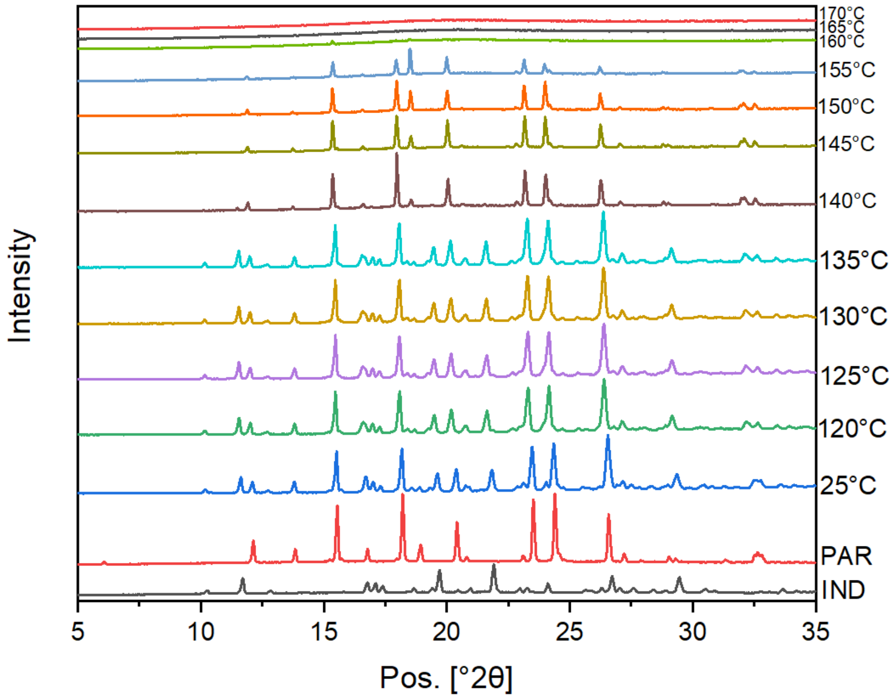

30% IND – 70% PAR

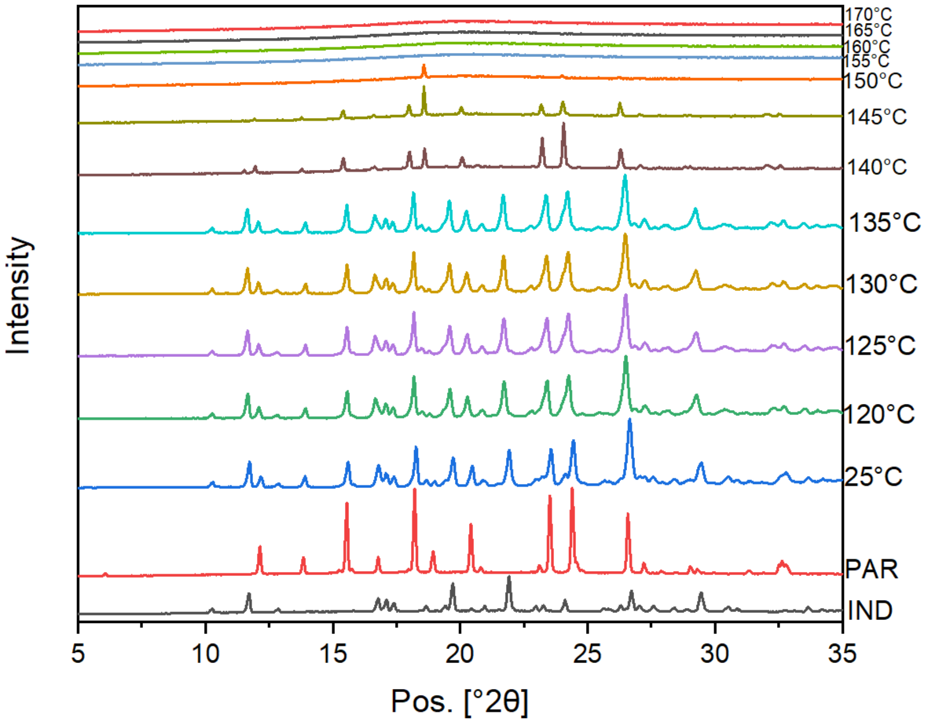

70% IND – 30% PAR

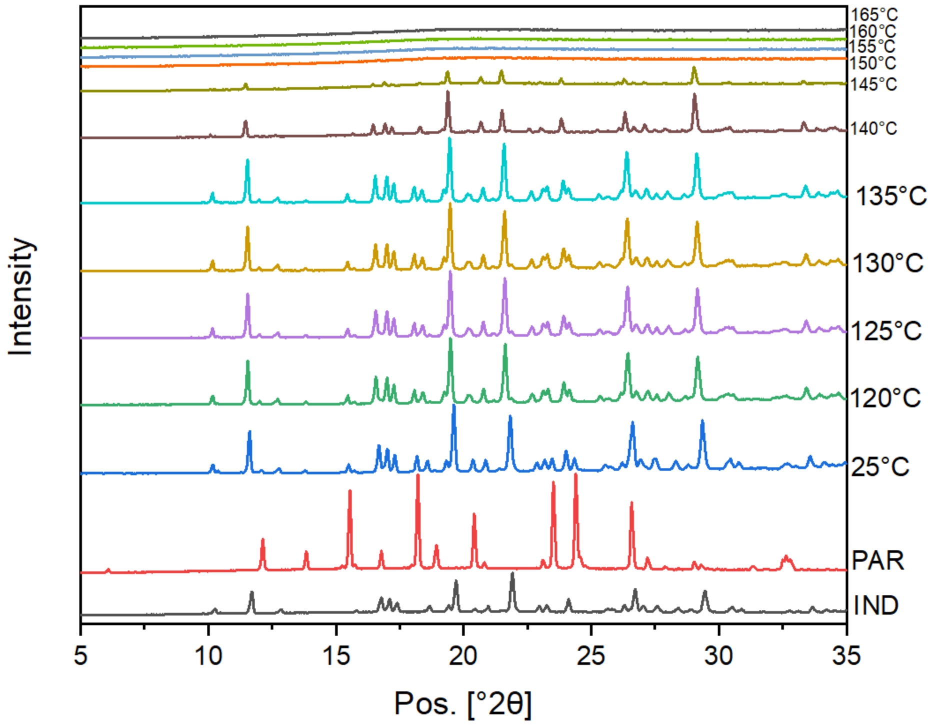

80% IND – 20% PAR

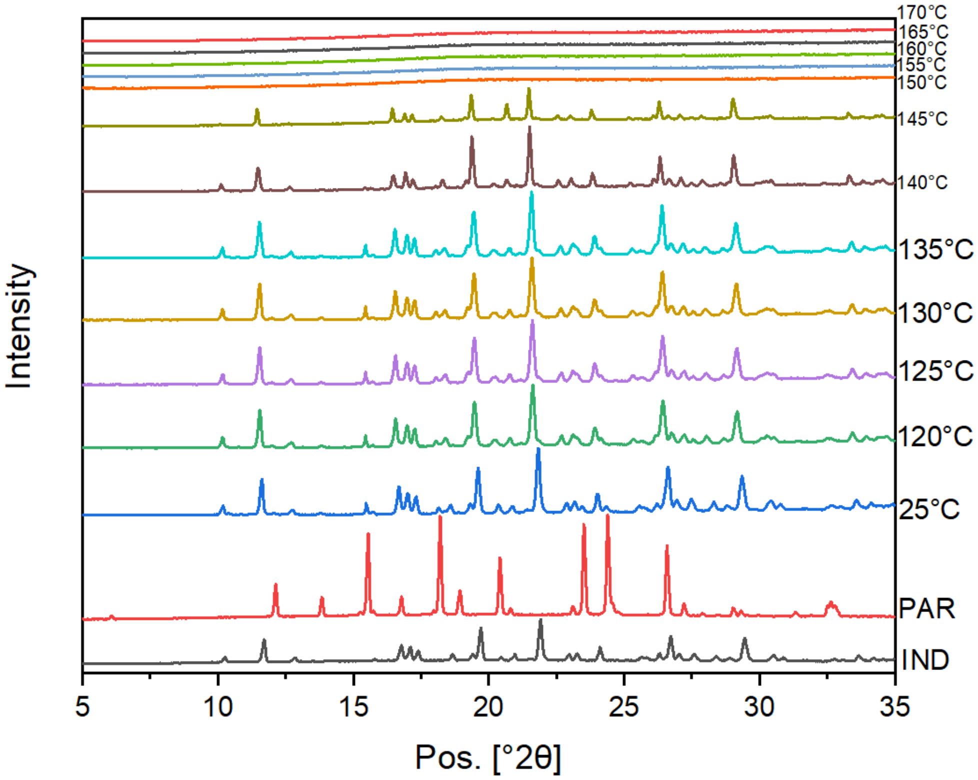

90% IND – 10% PAR

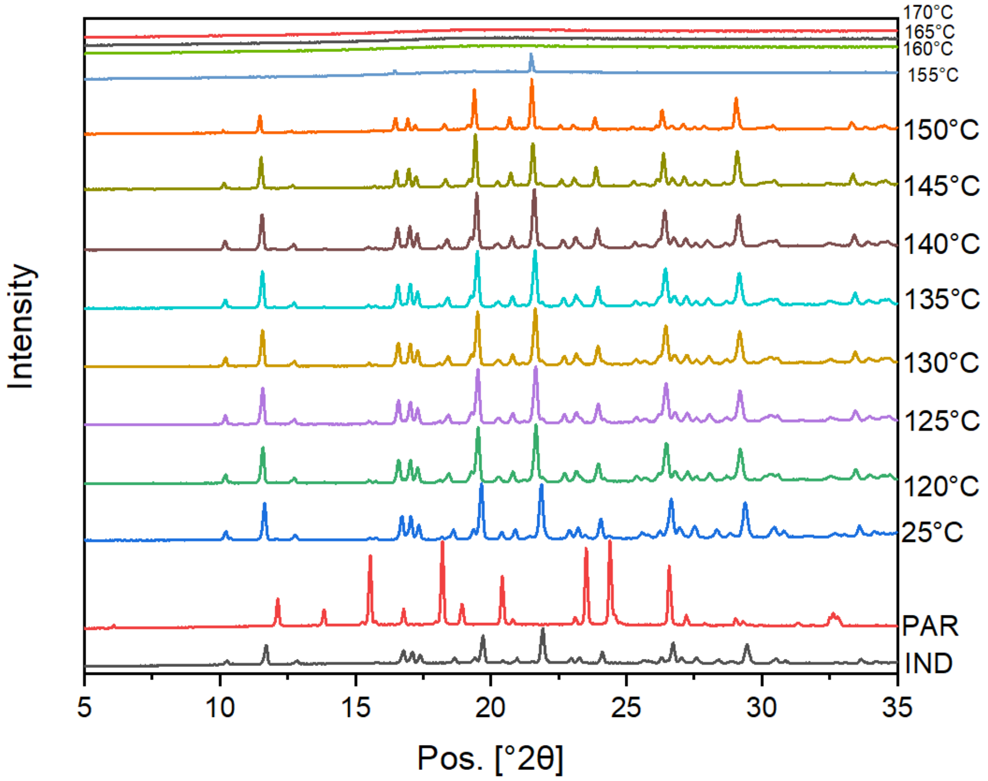
170°C
165°C
160°C
155°C
150°C
145°C
140°C
135°C
130°C
125°C
120°C
25°C
PAR
IND
Intensity
5
10
15
20
25
30
35
Pos. [°2θ]

Figure S2. Comparison of the theoretical Tg (calculated with the Gordon–Taylor equation) and the experimental Tg (obtained from DSC results) of IND – PAR co-amorphous systems at different mixing ratios (from 90% IND – 10% PAR to 10% IND – 90% PAR (mol/mol)). The Tg of amorphous IND and amorphous PAR were obtained from DSC measurements.

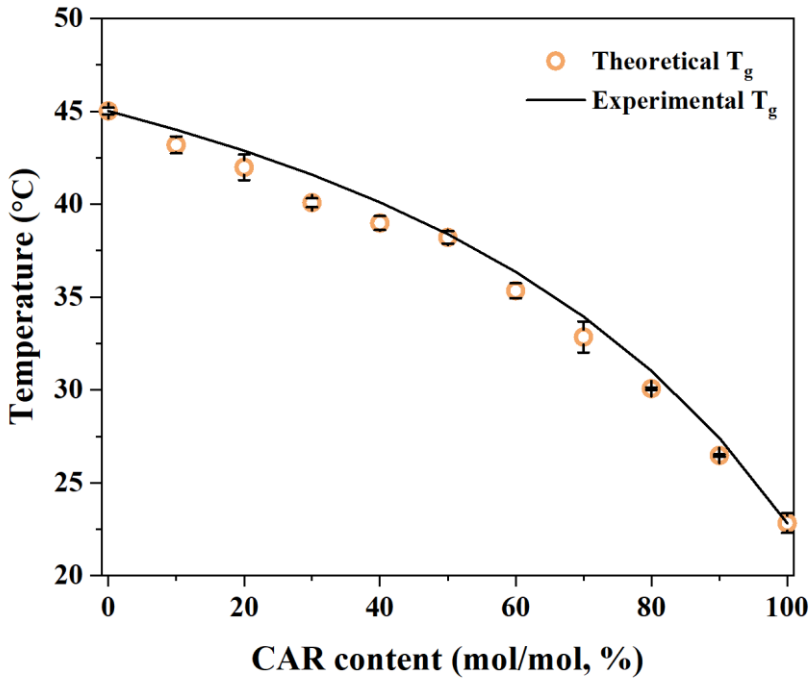

Figure S3. Stability test results of IND – PAR CAMS with various mixing ratios (from 90% IND – 10% PAR to 10% IND – 90% PAR (mol/mol)). The XRPD diffractograms of crystalline IND and crystalline PAR are also shown in the figure. Black arrows indicate crystalline refections. "w" in the figure represents "week.".

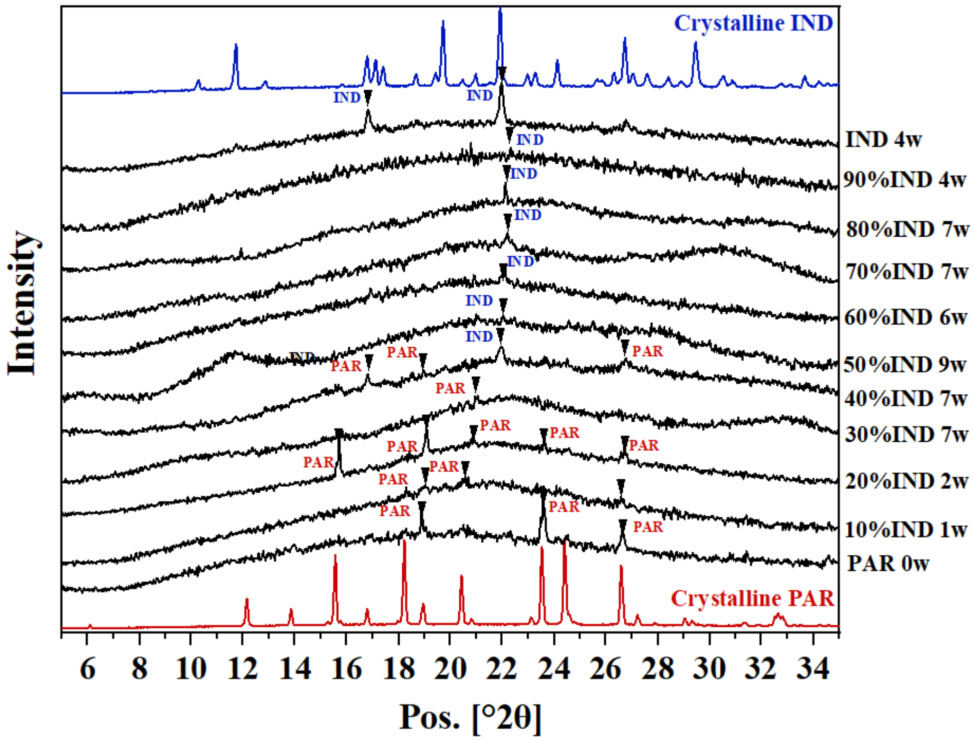

Figure S4. TGA results of CAMS and G-CAMS with different mixing ratios.

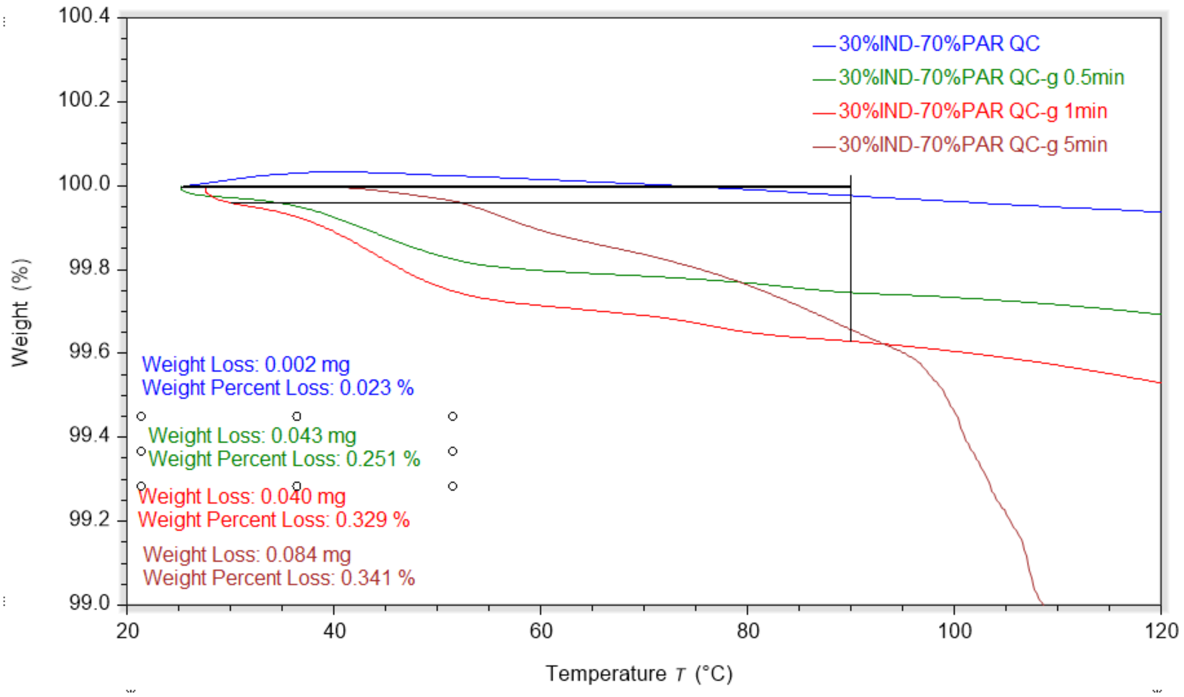
30%IND-70%PAR QC
30%IND-70%PAR QC-g 0.5min
30%IND-70%PAR QC-g 1min
30%IND-70%PAR QC-g 5min
Weight Loss: 0.002 mg
Weight Percent Loss: 0.023 %
Weight Loss: 0.043 mg
Weight Percent Loss: 0.251 %
Weight Loss: 0.040 mg
Weight Percent Loss: 0.329 %
Weight Loss: 0.084 mg
Weight Percent Loss: 0.341 %
Weight (%)
Temperature T (°C)

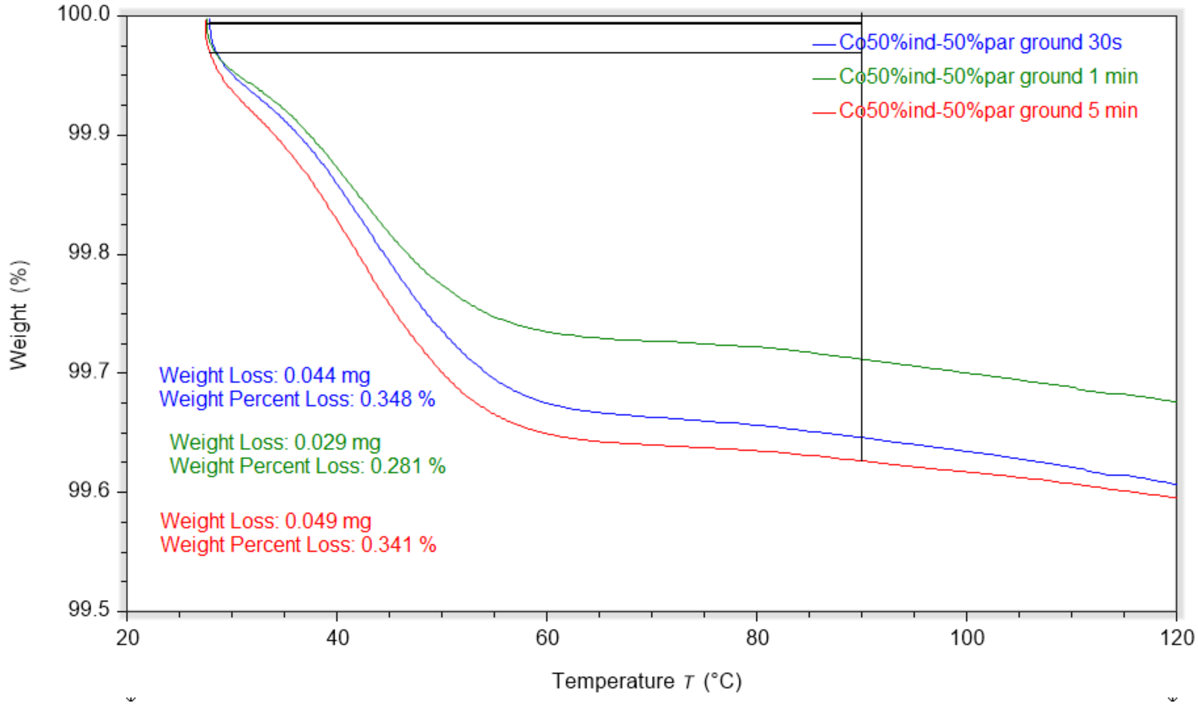
Co50%ind-50%par ground 30s
Co50%ind-50%par ground 1 min
Co50%ind-50%par ground 5 min
Weight Loss: 0.044 mg
Weight Percent Loss: 0.348 %
Weight Loss: 0.029 mg
Weight Percent Loss: 0.281 %
Weight Loss: 0.049 mg
Weight Percent Loss: 0.341 %
Weight (%)
Temperature T (°C)

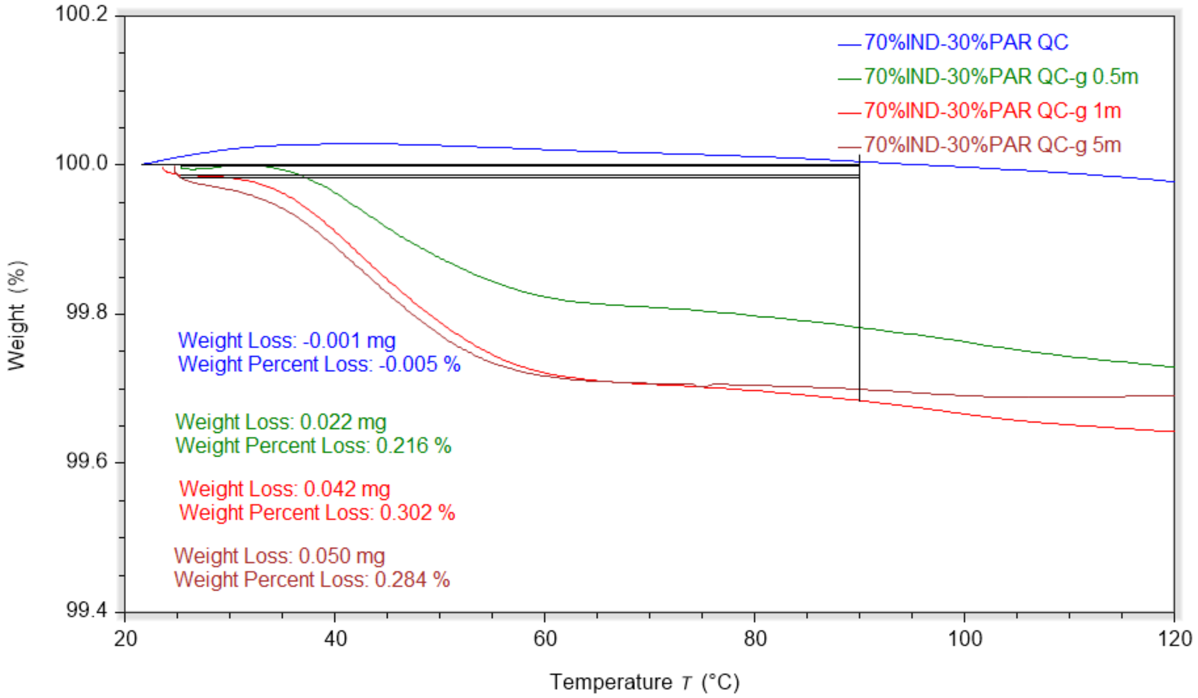

Figure S5. DSC and XRPD results of ground amorphous IND and ground amorphous PAR.

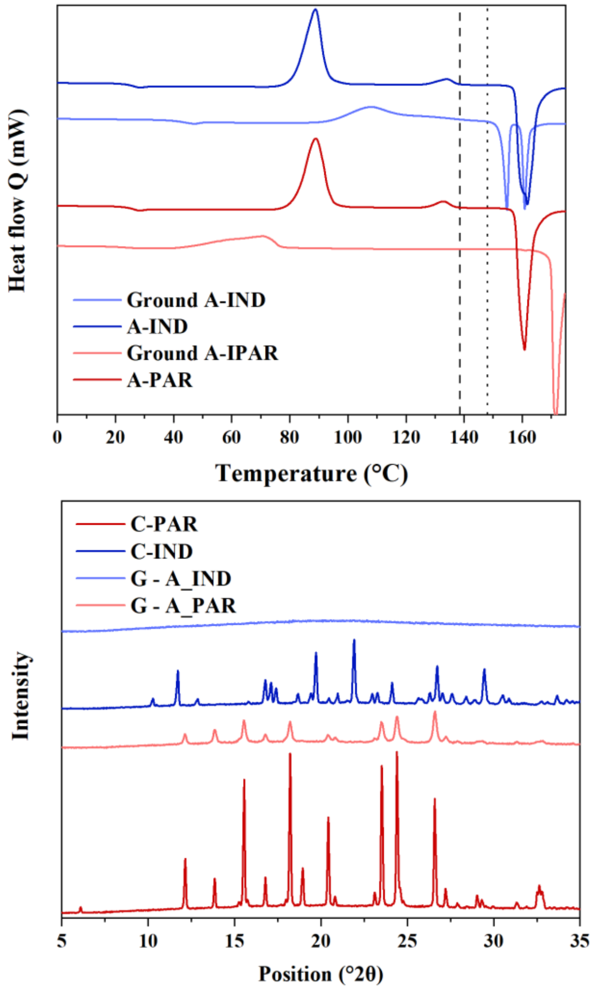

Figure S6. DSC thermographs of 30% IND – 70% PAR G-CAMS at different grinding times.

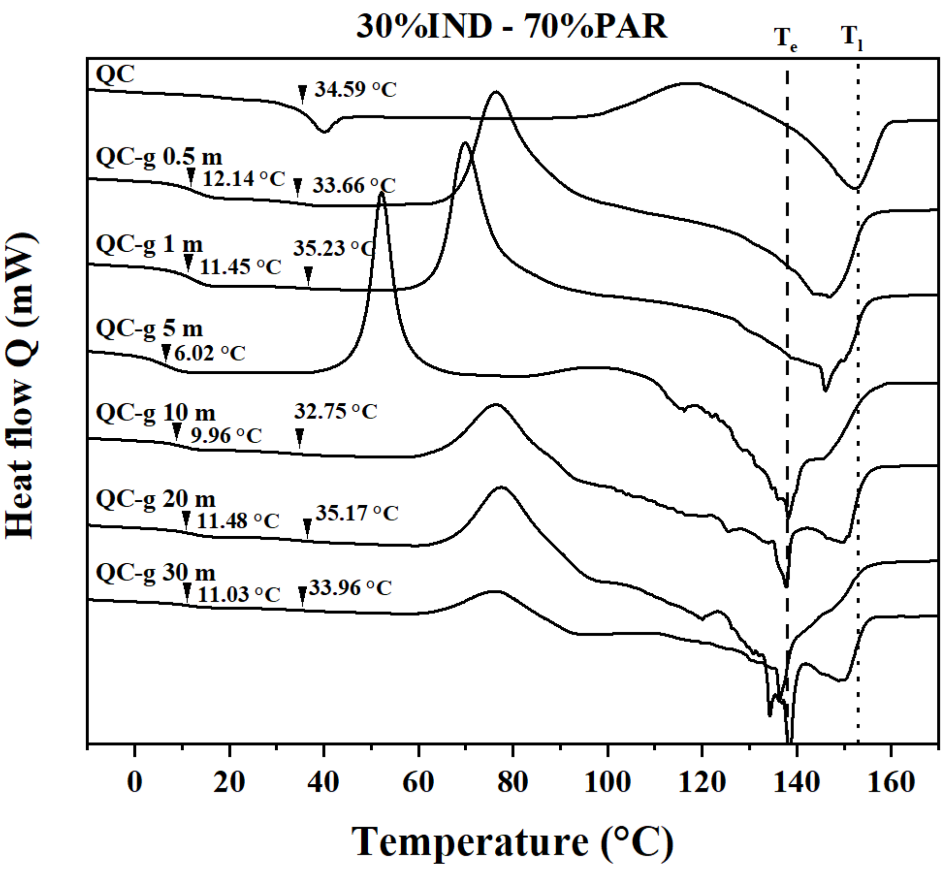

30%IND - 70%PAR
T_e
T_l
QC
34.59 °C
QC-g 0.5 m
12.14 °C
33.66 °C
QC-g 1 m
11.45 °C
35.23 °C
QC-g 5 m
6.02 °C
QC-g 10 m
9.96 °C
32.75 °C
QC-g 20 m
11.48 °C
35.17 °C
QC-g 30 m
11.03 °C
33.96 °C
Heat flow Q (mW)
0
20
40
60
80
100
120
140
160
Temperature (°C)
